# Serum miRNA Are Promising Biomarkers for the Detection of Early Hepatocellular Carcinoma after Treatment with Direct-Acting Antivirals

**DOI:** 10.3390/cancers11111773

**Published:** 2019-11-11

**Authors:** Devis Pascut, Luisa Cavalletto, Muhammad Yogi Pratama, Silvia Bresolin, Luca Trentin, Giuseppe Basso, Giorgio Bedogni, Claudio Tiribelli, Liliana Chemello

**Affiliations:** 1 Liver Research Center, Fondazione Italiana Fegato—ONLUS, AREA Science Park, Basovizza, 34149 Trieste, Italy; yogi.pratama@fegato.it (M.Y.P.); giorgiobedogni@gmail.com (G.B.); ctliver@fegato.it (C.T.); 2 Department of Internal Medicine—DIMED, University-Hospital of Padova, 35128 Padova, Italy; luisa.cavalletto@unipd.it (L.C.); liliana.chemello@unipd.it (L.C.); 3 Faculty of Medicine, Universitas Hasanuddin, Makassar 90245, Indonesia; 4 Laboratory of Onco-Haematology, Department of Women’s and Children’s Health, University of Padova, 35128 Padova, Italy; silvia.bresolin@unipd.it (S.B.);; 5 Istituto di Ricerca Pediatrica—Città della Speranza, 35127 Padova, Italy; 6 IIGM Torino and Pediatric Hemato-Oncology, 10126 Torino, Italy; giuseppe.basso@unipd.it

**Keywords:** HCC, microRNA, DAA, HCV, biomarkers

## Abstract

Direct antiviral agents (DAAs) have excellent efficacy against chronic hepatitis C virus (HCV) infection. Despite this strength, recent studies raised concerns about an unexpected hepatocellular carcinoma (HCC) occurrence rate after DAA therapy. In this exploratory case-control study, we evaluated the potential use of miRNAs as serum biomarkers for the detection of early HCC in DAA-treated patients. In the discovery phase, the circulating miRNome was assessed in 10 matched patients with (HCC+) or without HCC (HCC−) occurrence. Microarray analysis was performed before (T0) and after one month of the DAA therapy (T1). MiRNAs discriminating HCC+ and HCC− patients were validated in 60 samples by means of RT-qPCR. We estimated the time-averaged difference of a given miRNA between HCC+ and HCC− patients using a bootstrapped random-effect generalized least square regression model (RE-GLS). At T0, miR-1207-5p, miR-1275, miR-3197, miR-4443, miR-3178, miR-483-5p, miR-4706, miR-4793-3p and miR-1246 discriminated HCC+ from HCC− patients (*p* < 0.05). At T1, only miR-1180-3p, miR-1228-3p, miR-4329 and miR-4484 (*p* < 0.05) discriminated HCC+ from HCC− patients. The subsequent validation phase identified miR-3197 as changing with both disease and time. Our results suggest that patients might be already committed to HCC occurrence before DAA therapy. MiR-3197 shows some potential for the identification of patients at risk of HCC during DAA treatments.

## 1. Introduction

Worldwide, hepatocellular carcinoma (HCC) represents the fourth cause of cancer-related deaths [1]. Chronic hepatitis C virus infection (HCV) is one of the most common risk factors for HCC due to its direct viral oncogenic activity and through the establishment of chronic inflammation and fibrosis [2].

The availability of an effective therapy with direct-acting antivirals (DAAs) for HCV infection has dramatically increased the probability of viral eradication, with an efficacy up to 90% with respect to IFN-based schedules [3,4]. The excellence of the current safety profile leads to extensive use of DAAs in a large number of patients, previously excluded from IFN-based therapies, such as elder patients and those with advanced cirrhosis [5,6,7]. Multiple long-term follow-up studies have reported a reduced risk of HCC development after IFN-based treatments [8]. Despite expectations, recent studies raised concerns about the persistence of HCC risk after DAA therapy in patients with cirrhosis [9,10,11,12]. In addition, tumors occurring after DAA treatments were described as more aggressive, with multifocal and metastatic involvement [13,14]. Opposite conclusions were reached from multiple large cohort studies where the sustained virological response (SVR) was associated with a reduced risk of HCC [7,15] without any observation of more aggressive phenotypes [7]. Although the incidence of HCC was lower in patients with SVR compared to non-responders, a residual risk of tumor development exists, especially in patients with cirrhosis [16,17]. This is the reason why the EASL guidelines recommend a post-SVR surveillance of all patients with advanced fibrosis and cirrhosis [18].

Most studies so far pointed out the need for developing model-based evaluations of the risk of HCC in DAA-treated individuals using surrogate serum markers (i.e., levels of AFP and albumin, platelet count and APRI) and host characteristics (age, gender, comorbidities and presence of cirrhosis and DAA-resistance) [7,19]. For instance, in a recent study, post-treatment levels of the serum *Wisteria floribunda* agglutinin positive Mac-2 binding protein (WFA+M2BP) were used to predict HCC occurrence after viral eradication in patients with fibrosis [20]. In a case report, Ono and collaborators reported that the persisting risk of HCC after SVR in a patient with cirrhosis could be evaluated by a tissue transcriptome profile [21]. Thus, suggesting a biomarker-based cancer risk prediction model is a viable option in DAA treatments. The present study opens new perspectives in exploring serum surrogate biomarkers to accurately identify high-risk patients. Nowadays, the availability of new RNA sequencing technology has led to the discovery of thousands of non-coding RNA (ncRNA) genes. Among these RNAs, microRNAs (miRNAs) are a class of highly conserved short-non coding RNAs with 18 to 25 nucleotides participating in posttranscriptional gene regulation [22]. They are considered promising candidates for serving as non-invasive biomarkers because they are generally available and relatively stable in serum [23]. Numerous studies have evaluated the role of miRNAs as circulating biomarkers for HCC in different clinical settings [24,25,26]. Since late diagnosis is limiting the therapy options for the patients, making HCC one of the deadliest cancers worldwide, the use of circulating biomarkers for early diagnosis, or for predictive models, represents a relevant unmet clinical need for liver oncology.

The present exploratory study was designed to evaluate the potential use of miRNAs as serum biomarkers for HCV-related HCC in patients undergoing DAA therapy. In addition, the study aimed for evaluating the influence of DAAs on de novo HCC development by comparing the circulating miRNome profile before and after DAA therapy in patients with and without development of HCC.

## 2. Results

### 2.1. Characteristics of the Study Population

A total of 150 patients with HCV-related liver cirrhosis treated with DAA from January 2015 to December 2016 were the study population. They had a mean (SD) age of 59 (11) years and were mostly males (55%); 70% of them had Child A5 cirrhosis, 66% had HCV genotype 1 and 86% were treated with Sofosbuvir.

Twenty patients who developed HCC (HCC+ or “cases”) during a mean (SD) follow-up of 5.3 (4.8) months from the end of therapy (EOT) were matched according to age, gender, HCV genotype and therapy schedule to 20 patients who did not develop HCC (HCC− or “controls”) with a mean (SD) follow-up of 32 (±9) months from EOT. Table 1 gives the features of all the study subjects, separated into a discovery and validation group. There were neither clinically relevant nor statistically significant differences between the discovery and validation groups. Serum samples from patients were collected and analyzed at two time points: Before (T0) and at one month (T1) after DAA therapy, to explore changes in the miRNA expression profile.

### 2.2. Changes in Circulating miRNA after DAA Therapy

We examined the expression levels of the circulating miRNome in the serum of 10 patients with chronic HCV infection that underwent DAA therapy. MiRNA expression profiles were generated by using gene array miRNA 3.0. According to the Absent/Present calling of the Affymetrix algorithm, among the 1734 miRNAs analyzed, 1466 were absent in our samples, while 265 miRNAs were considered as expressed in both T0 and T1 (Figures S1a,b and S2a,b). This number is in agreement with a previous study performed in another group of patients, where from 130 ng of purified serum small RNAs we were able to detect 274 different species of mature miRNAs [27]. In order to verify the influence of the DAA treatment on the circulatory miRNome, we compared T0 and T1 profiles in cases and control groups. Interestingly, comparing T0 vs. T1 expression profiles, we noticed that DAAs and/or virus load variations induced significant miRNA perturbations in both groups (Table S1 and Figure S3). MiR-92a, miR-1225-5p and miR-4253 have the highest variation during the DAA treatment with an up-regulation greater than 10 FC at T1. On the contrary, miR-320d and miR-320a were significantly decreased after one month of therapy (Table S1).

However, when exploring the changes from T0 to T1, we noticed a different pattern for the two groups of patients (Tables S2 and S3). Interestingly, miR-1225-5p was highly increased from T0 to T1 only in patients without HCC. While, miR-483-5p and miR-3178 were significantly decreased only in patients with HCC (Tables S2 and S3).

MiR-297, miR-3180-3p, miR-1268, miR-4701-3p, miR-4417, miR-1910 and miR-459 have a similar pattern of changes in the two groups. Interestingly the liver-specific miR-122 decreased after DAA treatment (FC = 5.25), although not reaching statistical significance (*p* = 0.06).

### 2.3. MicroRNA Screening in the Discovery Cohort, Identification of Predictive miRNA

**Figure 1:**
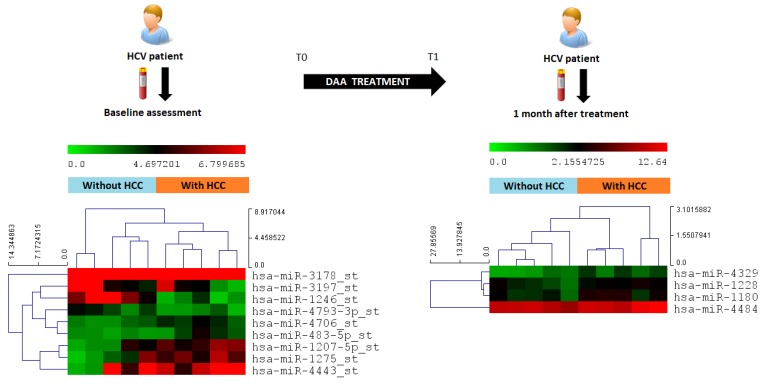
Serum samples were collected from patients before and after DAA treatment initiation. Circulating miRNome profiles were analyzed by miRNA array at both times. Statistically significant miRNAs were included into the heatmap with the pseudocolor scale underneath. Unsupervised hierarchical clustering is used to order samples and miRNAs; the log2-transformed microarray signal was considered. The sample tree with optimized leaf-ordering is drawn using Euclidean distances and average linkages for cluster-to-cluster distances.

In order to determine the differences in the circulating miRNA expression at each time point for the two groups of patients analyzed in the discovery phase, we performed a pairwise comparison of the miRNA array results. At T0, we identified 9 miRNAs, miR-1207-5p, miR-1275, miR-3197, miR-4443, miR-3178, miR-483-5p, miR-4706, miR-4793-3p and miR-1246, significantly and differently expressed between the group with HCC and without HCC (ANOVA *p* < 0.05) (Figure 1).

A different miRNA profile distinguishing the two groups was identified at T1, including miR-1180-3p, miR-1228, miR-4329 and miR-4484 (Figure 1).

### 2.4. Differential miRNA Expression Profiles and Identification of miRNA Biomarker Candidates 

We used the RT-qPCR assays to confirm the expression of 13 miRNAs candidates that were differently expressed (*p* < 0.05) in the discovery cohort at T0 and T1. We assessed the miRNA expression in other 60 matched samples (30 HCC+ and 30 HCC−). Among the selected miRNAs, we were not able to confirm by RT-qPCR assays the expression of miR-4706, miR-4793-3p and miR-4329. In addition, the variance among samples of miR-1275 was extremely low (0.35) compared to the other samples that were discarded for subsequent analysis. Due to the lack of agreement among studies reporting miRNA expression data, we decided to perform the analysis on both the Cq and ΔΔCq data of the selected miRNA reported in Table 2.

**Figure 2:**
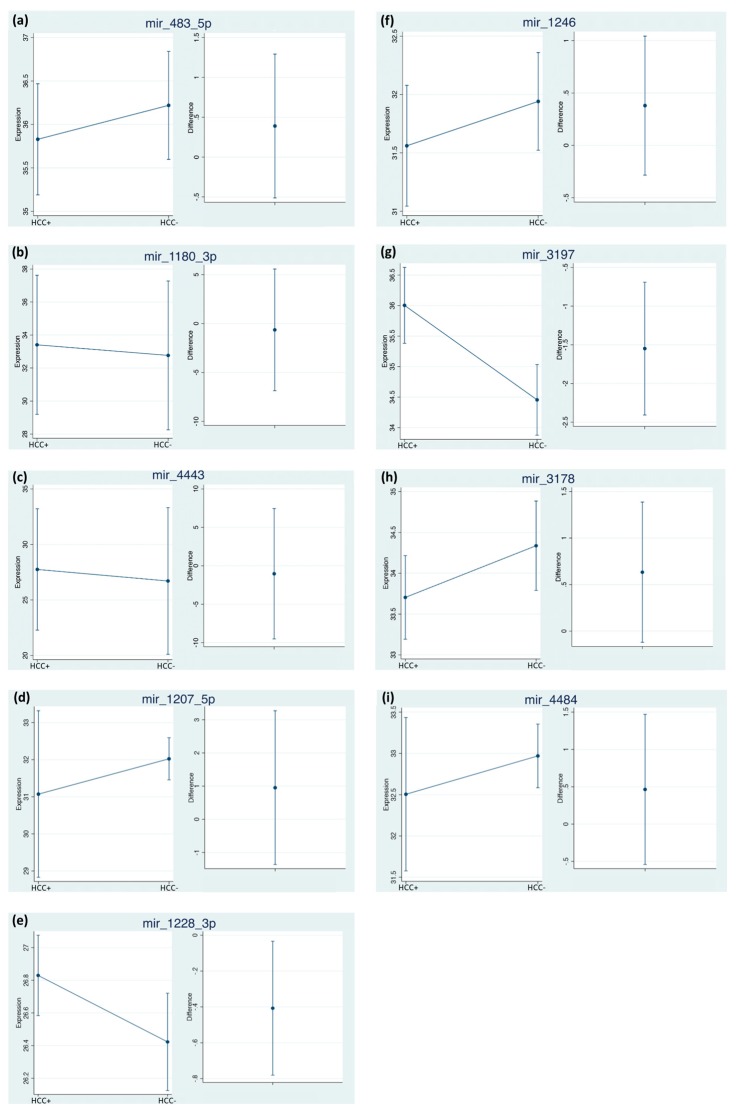
Time-averaged differences of miRNAs between subjects HCC+ vs. HCC−. The estimates were obtained using bootstrapped random-effect generalized least square regression (RE-GLS) calculated on the Cq expression values. Internal cross-validation was performed using bootstrap on 1000 samples with replacement (see statistical analysis for details). The difference is calculated as (HCC+ minus HCC−), values showing 95% CI crossing 0 are not statistically significant at the 0.05 level.

When estimating the time-averaged difference of the two-repeated measures for each miRNA between subjects with HCC vs. without HCC, we identified significant changes, considering both times in miR-3197 and miR-1228-3p, both showing an increased Cq value in the group with HCC (Figure 2e,g). Interestingly, miR-3197 showed the highest Cq differences between the two considered groups.

**Figure 3:**
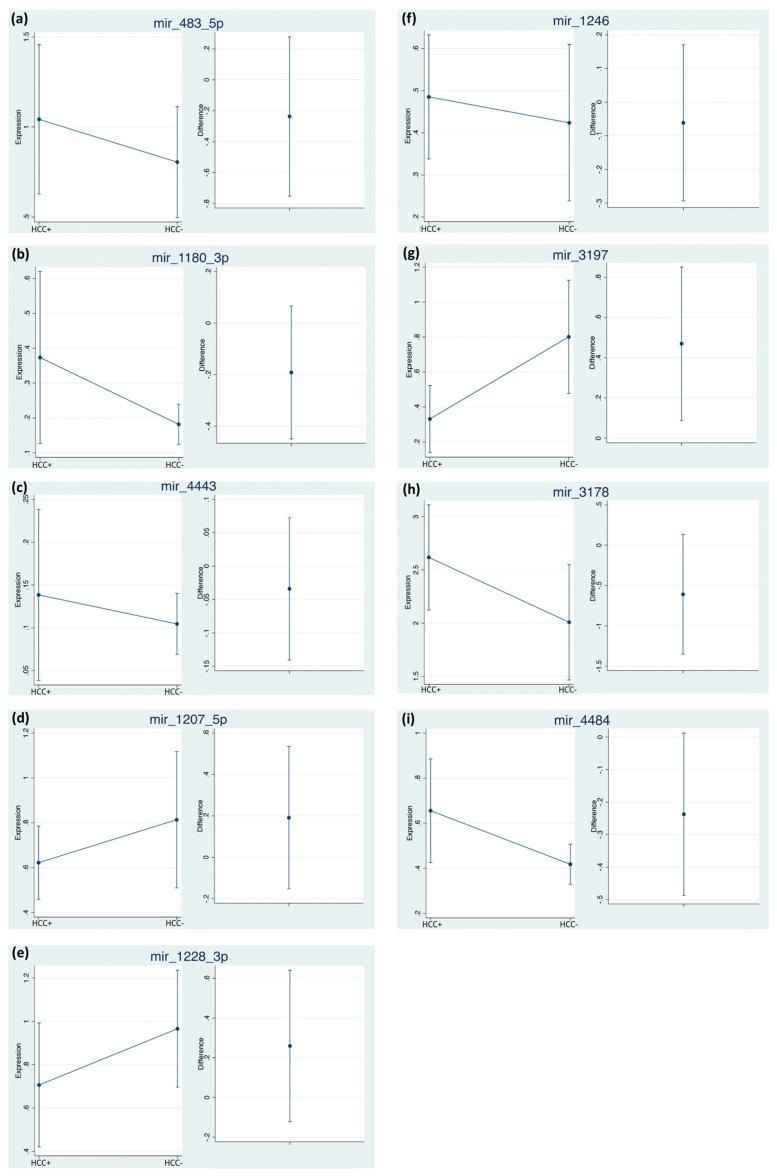
ΔΔCq time-averaged differences of miRNAs between subjects HCC+ vs. HCC−. The estimates were obtained using bootstrapped random-effect generalized least square regression (RE-GLS). Internal cross-validation was performed using bootstrap on 1000 samples with replacement (see statistical analysis for details). The difference is calculated as (HCC+ minus HCC−), values showing 95% CI crossing 0 are not statistically significant at the 0.05 level.

Despite not being statistically significant, miR-483-5p, miR-1246, miR-3178, miR-1207-5p and miR-4484 showed a higher expression in patients with HCC. While, miR-1180-3p and miR-4443 had no differences both in terms of time and presence of the disease (Figure 2b,c). By considering the normalized data, we confirmed the statistical relevance of miR-3197 (Figure 3g), being 2.42 times lower in HCC patients. The expression of miR-3197 was further confirmed through qRT-PCR in the discovery cohort (Figure S6). MiR-1228 did not reach statistical significance anymore after normalization.

### 2.5. MiR-3197 as Diagnostic Biomarker

**Figure 4:**
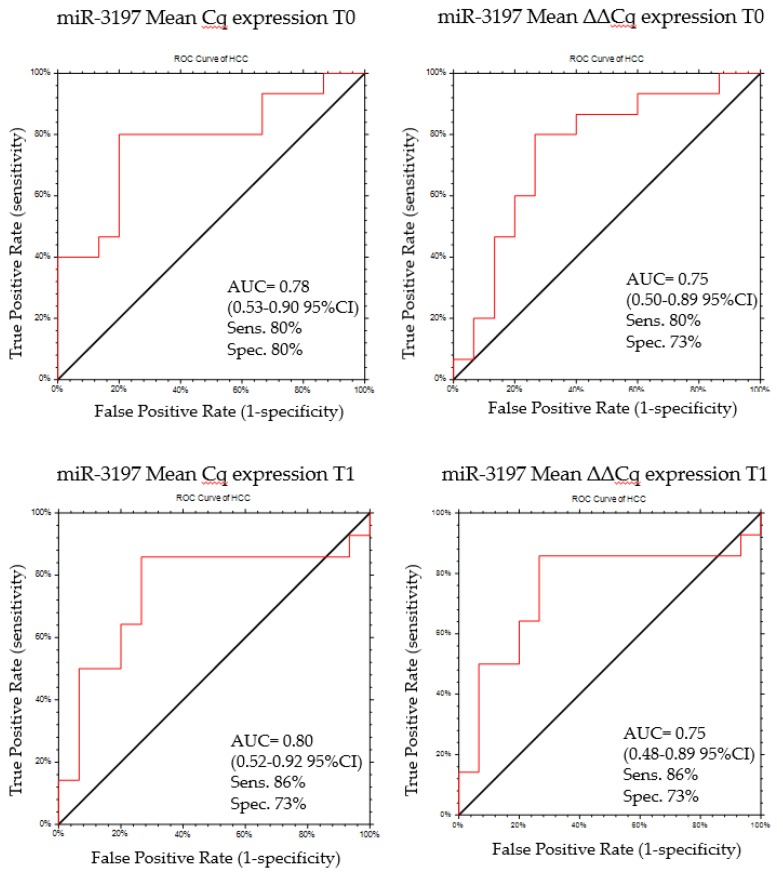
ROC curve for miR-3197 discriminatory potential between patient HCC+ and HCC− at T0 and T1 using both normalized and non-normalized data.

MiR-3197 was confirmed as a potential biomarker candidate from both normalized and non-normalized data analysis. In order to validate the discriminatory potential of miR-3197, we calculated the area under the curve (AUC) of the receiver operator characteristic (ROC) curve at T0 and T1. Using a cut-off of Cq ≥ 35.07, miR-3197 can distinguish patients with HCC at T0 with a sensitivity and specificity of 80% and 80%, respectively (AUC = 0.78 (0.53–0.90 95% CI), *p* = 0.0009) (Figure 4). Similar performances were obtained when using the ΔΔCq values. Using a cut-off of ΔΔCq ≤ 0.45, miR-3197 can distinguish patients with HCC at T0 with a sensitivity and specificity of 80% and 73%, respectively (AUC = 0.75 (0.50–0.89, 95% CI), *p* = 0.004) (Figure 4). At T1 using a cut-off of Cq ≥ 34.69 the sensitivity and specificity of miR-3197 in distinguishing cases vs. controls increased to 86% and 73%, respectively (AUC = 0.80 (0.52–0.92, 95% CI), *p* = 0.0009) (Figure 4). The same values of sensitivity and specificity were obtained when using the ΔΔCq, at a cut-off ΔΔCq ≤ 0.45, with an AUC of 0.75 (0.48–0.89 95% CI, *p* = 0.007).

The less strong evidences for miR-1228 were confirmed by the ROC curve and cut-off analysis. At T0 the sensitivity and specificity of miR-1228-3p in distinguishing patients with or without HCC were 80% and 60%, respectively (AUC = 0.70 (0.45–0.85, 95% CI), *p* = 0.019), with a cut-off ≥ 26.82. After normalization, the AUC decreases to 0.60 (0.35–0.77, 95% CI), with a sensitivity and specificity of 60% and 53.3%, with a cut-off ≤ 0.60. At T1 the sensitivity and specificity of miR-1228-3p in distinguishing patients with HCC from the patients without HCC were 86% and 79%, respectively (AUC = 0.70 (0.45–0.85, 95% CI), *p* = 0.019), at a cut-off of ≥ 26.57 (AUC = 0.69 (0.42–0.85 95% CI, *p* = 0.03). Once again, after normalization the AUC reduces to 0.63 (0.37–0.80 95% CI), with a sensitivity and specificity of 79% and 66%, respectively, determined for a cut-off ≤ 0.66.

## 3. Discussion

Hepatocellular carcinoma is the leading complication of HCV-related cirrhosis and although direct DAAs showed an efficacy in viral eradication up to 90%, they do not abolish the risk of HCC appearance. These observations highlight the importance of the identification of patients at risk of developing HCC after DAAs for an individual surveillance program [18]. Recently, Nagata and collaborators predicted the HCC occurrence in patients with fibrosis by measuring the post-treatment levels of the serum WFA+M2BP [20]. In a case report study, Ono et al. showed the capability of the tissue transcriptome profile to identify a persistent risk of HCC after SVR in a patient with cirrhosis [21]. These issues open new perspectives in the use of biomarker-based models to identify cancer risk patients after DAA treatments.

In this study, the circulating miRNome profiles of patients who developed HCC were compared before (T0) and during (T1) DAA treatment and matched to controls without the appearance of a tumor during follow-up, to address the possible use of miRNAs as a signature for early diagnosis of HCC occurrence after DAA therapy.

In the discovery phase, miRNA array data of 10 patients (5 pair-matched with and without HCC development) were analyzed at T0 and T1. Interestingly, the overall effect of DAA therapy on the circulating miRNome had a relevant impact on both groups with very large variations (greater than 10 FC) involving miR-92a, miR-1225-5p and miR-4253. In particular, miR-1225-5p was upregulated from T0 to T1 only in patients without HCC development. This is in line with recent findings of an anti-cancer effect of miR-1225-5p in different cancer types [28,29]. The comparison of T0 miR-profile among patients with and without appearance of HCC showed statistical difference for miR-1207-5p, miR-1275, miR-3197, miR-4443, miR-3178, miR-483-5p, miR-4706, miR-4793-3p and miR-1246 (*p* < 0.05). These differences could support the hypothesis of a pre-existent commitment of some patients to HCC before the DAA initiation. Currently, there is no clear evidence of any contribution of DAAs in the development of tumors in predisposed patients. Faillaci and collaborators suggested that the DAA-mediated increase of VEGF might contribute to tumor growth in cirrhotic subjects with already over-expressed Angioprotein-2 in liver tissues [30]. Other hypotheses claim the reduction of immune surveillance as a possible enhancer of tumor development in patients with a compromised liver [31,32]. Indeed, several immune mediators have been found to be differently regulated in patients who developed HCC after DAA treatments, compared with matched controls with no HCC evidence [33]. We believe that the dramatic reduction of the viral load may have an impact mainly in the liver and immune system, and those perturbations are somehow evidenced by changes in miRNA expression profiles. In addition, Waring and collaborators described a reduction in miR-122, a liver specific miRNA having a well-documented role in HCV infection [34], in patients achieving an SVR [35]. These observations are in line with our preliminary results where miR-122 was downregulated after the DAA treatment.

Considering the difference observed between cases and controls at T1, other miRNAs, such as miR-1180-3p, miR-1228, miR-4329 and miR-4484, significantly distinguished the two groups, being all up-regulated in patients developing HCC within 12 months after the stop of the DAA therapy.

Taking into account the role in cancer [36,37,38,39,40,41,42,43,44,45,46] of the miRNAs identified in the discovery phase, we further investigated the expression by RT-qPCR of the 13 miRNA candidates in a cohort of 60 matched samples, (30 HCC+ vs. 30 HCC−) obtained at T0 and T1. The time difference of the mean obtained from repeated measures of the selected miRNAs in HCC+ vs HCC− patients, confirmed by bootstrap cross validation analysis on 1000 samples, evidenced the significant changes in miR-3197 and miR-1228-3p when analyzing the raw Cq expression. By normalizing the data, only miR-3197 was confirmed as a potential serum biomarker for the identification of patients at risk for developing HCC. The better performances of miR-3197, compared to miR-1228-3p, were also evidenced by the ROC curves analysis, in which miR-3197 had higher AUC values with higher sensitivity and specificity in distinguishing patients with HCC from patients without HCC, at both times.

Based on these evidences, miR-3197 could represent a serum biomarker candidate for the identification of patients at risk after DAA treatment protocols, particularly in HCV-related liver cirrhotic patients. Considering the high rate of HCC observed in our cohort (13.3%), this biomarker could be used for a better surveillance program, particularly in light of some evidences showing a more aggressive HCC phenotype in DAA-treated patients [13,47].

In addition, by comparing HCC serum vs. total blood samples, we found miR-3197 expressed only in serum suggesting an extra-blood origin for this miRNA, possibly cancer tissue. Indeed, miR-3197 was found down-regulated in tissue of patients with a history of recurrent HCC after liver transplantation [40].

To our knowledge, this is the first study assessing the serum miR-3197 expression in relation to HCC occurrence. At present, due to the general lack of information about this miRNA, and based on the putative targets found in miRTarBase, we can speculate an activity in cell growth, proliferation and cell adhesion by involving IGF1, NDEL1, TNFRSF13C and FBXL18 pathways. However, further experimental validations are needed to confirm this hypothesis.

## 4. Materials and Methods

### 4.1. Patient Characteristics

We conducted a real-life-practice observational study on prospective out-patients treated for chronic HCV infection with DAAs from January 2015 to December 2016. Patient recruitment was carried out in Padua University Hospital liver centers, and the major objectives of the study were (a) to investigate the development of de novo liver cancer after DAA therapies and (b) to identify miRNA profiles that predicts early emergence of HCC after DAAs. Thus, our study has been focused on comparison of 40 matched cirrhotic subjects with and without development of HCC after DAA therapy. A total of 206 patients were treated with DAAs and, according to the exclusion criteria, 36 patients with F3 fibrosis staging (no cirrhosis), 13 with a previous history of HCC, 5 without an imaging check close before therapy initiation and 2 drop-outs were excluded. One hundred and fifty patients were included, and among these, 20 subjects with development of HCC within a mean time of 6 + 4 months after stopping DAA therapy. The patients with HCC were compared to the 20 selected, among the remaining 130 patients without development of HCC, by a software for one-to-one pair-matched according to age, gender, HCV genotype and therapy schedule. All subjects gave their informed consent to participate in the study that was conducted according to the rules of Helsinki declaration and approved by the local ethics committee Prot N° 3386/AO/14, approved on 12/01/2015 and Cod. NRC AOP0357, being part of a Regional Survey Program within HUB centers on antiviral therapy for chronic hepatitis and cirrhosis associated to HCV infection.

### 4.2. Study Design

**Figure 5:**
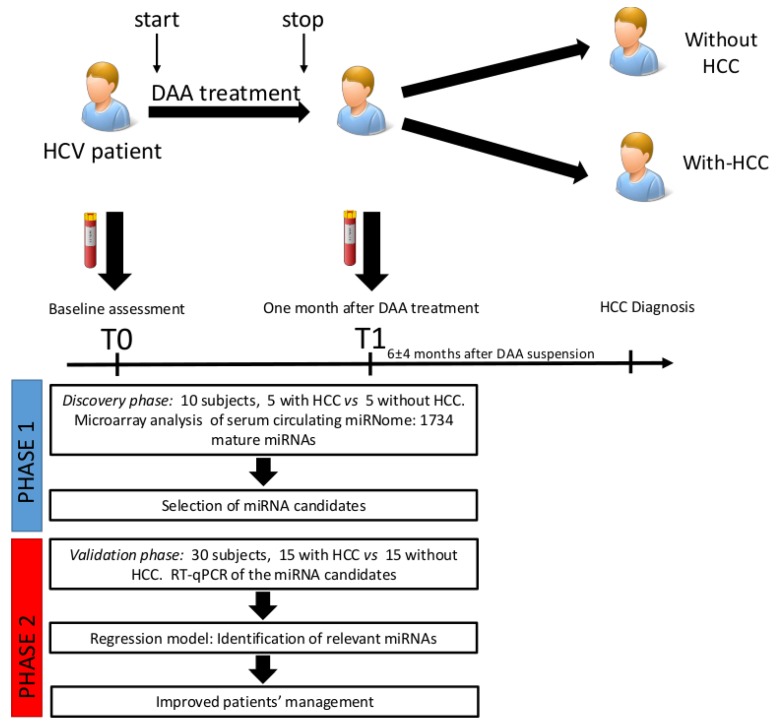
General scheme of the miRNA profiling study design. In the discovery phase 10 patients were enrolled and analyzed through a Genechip miRNA 3.0 array. Patients were divided into two groups, with-HCC (subjects developing HCC after DAA treatment) and without-HCC (subjects not developing HCC after DAA treatment). Samples were analyzed at T0 and T1. Subsequently, miRNA candidates were assessed by qRT-PCR in 30 patients (validation cohort). MiRNA biomarkers were selected by estimating the time-averaged difference of a given miRNA between subjects with HCC vs. without HCC using a bootstrapped random-effect generalized least square regression model (RE-GLS).

This study was organized as follows: (1) Discovery phase (Phase 1). Serum samples from 10 patients treated with DAAs (5 pair-matched between patients with and without development of HCC) were analyzed through microarray profiling. Analysis was performed at two time points: Before DAA treatment (T0) and after 1 month of DAA treatment (T1) to explore changes among miRNA in the two groups. One-way analysis of variance (ANOVA) tests were used to determine gene expression differences among subjects who developed HCC (HCC+) vs. those who did not (HCC−) (Figure 5). Multiple testing corrections were performed with the Benjamini–Hochberg method. In the discovery cohort, a panel of candidate circulating miRNAs was selected based on the differential fold of change (cut-off: ±1.5) and significance level (*p* < 0.05). (2) Validation phase (Phase 2). The miRNA candidates selected in the discovery phase were tested by quantitative Real Time PCR (RT-qPCR) in an independent cohort of 30 patients treated with DAAs (15 pair-matched between patients with and without development of HCC) at T0 and T1. Analysis was performed by estimating the time-averaged difference of a given miRNA between subjects with HCC vs. without HCC using a bootstrapped random-effect generalized least square regression model (RE-GLS).

### 4.3. Serum Collection and RNA Extraction

Serum samples were obtained from 10 mL of whole blood collected in sterilized tubes and centrifuged at 3000 r/min in a refrigerated centrifuge. Supernatants were transferred in 1 mL Eppendorf tubes and subsequently frozen at −80 °C for long-term storage. Small RNAs were isolated from 300 uL of serum using the miRCURY™ RNA Isolation Kits—Biofluids (Exiqon, Vedbaek, Denmark). MicroRNAs were quantified in a Qubit^®^ 2.0 Fluorometer (Thermo Fischer Scientific, Waltham, MA, USA) by using the Qubit microRNA Assay Kit (Thermo Fischer Scientific) following the manufacturer’s instructions.

### 4.4. Microarray Profiling 

130 ng of purified small RNAs were labelled with the FlashTag™ Biotin HSR RNA Labeling Kit (Affymetrix^®^, Thermo Fischer Scientific) and hybridized on Genechip miRNA 3.0 (Thermo Fischer Scientific) containing 1734 human mature miRNAs. The array cartridges were processed on an Affymetrix Fluidic Station 450 and scanned on an Affymetrix GeneChip 3000 7G. The robust Multichip Analysis (RMA) algorithm was used to derive CEL file probe-level hybridization intensities at the gene expression levels.

### 4.5. Quantitative Real Time PCR (RT-qPCR)

Microarray results were further validated by using Quantitative Real Time PCR (RT-qPCR) in the second independent cohort of 30 patients treated with DAAs. The subjects were analyzed at T0 and T1 (Figure 1). Ten nanograms of small RNAs were reverse transcribed by using the qScript microRNA cDNA Synthesis Kit (Quantbio, Beverly, MA, USA) according to manufacturer’s instructions. The RT-qPCR was performed with the PerfeCTa SYBR^®^ Green SuperMix (Quantbio) in a CFX-96 thermal cycler (Bio-Rad Laboratories, Hercules, CA, USA) according to manufacturer’s instructions. The Cq for each miRNA was obtained by calculating the arithmetic mean average of triplicates in a 20 µL reaction. Cq values > 45 were considered as negative, and the melting point curves were observed for all assays to verify primer specificity. All the primers were from Sigma-Aldrich (Merck KGaA, Darmstadt, Germany). The relative quantification was obtained using the Pfaffl modification of the ΔΔCq equation, taking into account the efficiencies of individual genes and results were normalized to miR-1280.

### 4.6. Microarray Data Analysis

One-way analysis of variance (ANOVA) test were used to determine gene expression differences in the microarrays. Multiple testing corrections were performed with the Benjamini–Hochberg method and false discovery rate (FDR), and corrected *p*-values were calculated. The Absent/Present calling of the Affymetrix algorithm included in the Affymetrix Transcriptome Analysis Console was used to select the miRNAs considered as “present” in the analyzed samples. A heatmap with the pseudocolor scale underneath of the differentially expressed miRNAs were generated by using Mev 4.9.0 software. Unsupervised hierarchical clustering was used to order samples and miRNAs. The sample tree with optimized leaf-ordering was drawn by using Euclidean distances and average linkages for cluster-to-cluster distances.

### 4.7. Statistical Methods

We estimated the time-averaged difference of a given miRNA between subjects with HCC vs. without HCC using a bootstrapped random-effect generalized least square regression model (RE-GLS). The RE-GLS model used the given miRNA (continuous) as the dependent variable and time (discrete; 0 = time 1; 1 = time 2), HCC (discrete; 0 = no; 1 = yes) and a time * HCC interaction (discrete * discrete) as predictors. Using such a model, we used a specific contrast to estimate the time-averaged difference of the 2 repeated measures of the given miRNA in the group with HCC vs. the group without HCC. The random effect of the RE-GLS was assigned to the patient. Internal cross-validation was performed using bootstrap on 1000 samples with replacement. This is expected to correct for over-optimism and make the model more generalizable [48]. The analysis was performed considering both the raw Cq values and the ΔΔCq values. The receiver operating characteristic (ROC) curves were plotted to estimate the discriminatory potential of the miRNAs. Analyses were performed by using NCSS 11 Software (2016) (NCSS, LLC. Kaysville, UT, USA, ncss.com/software/ncss) and Stata 16.0 (Stata Corporation, College Station, TX, USA).

## 5. Conclusions

In this case-control exploratory study, we reported several findings concerning the HCC occurrence in DAA-treated patients. 

First, we observed important changes in circulating miRNome in all patients from T0 to T1, possibly reflecting a sort of perturbation of the hepatocyte environment and/or of immunological status, perhaps due to viral load variations and/or associated to DAA use. This should also explain the quick time for progression, the atypical tissue invasiveness and the resistance to multimodal therapies showed by patients who developed HCC. Secondly, we have identified a different miRNA panel characterizing patients with and without HCC at T0, before starting treatment, indicating a pre-commitment in development of tumors during DAA therapy. Third, we described miR-3197 as the HCC-related biomarker that can be used to monitor patients treated with DAAs, especially in populations at risk, such as those with cirrhosis, diabetes, alcohol abuse, older age, male gender and metabolic syndromes [7,16,17,49]. To our knowledge, this is the first case-control study with repeated measures to assess the circulating miRNAs in HCV patients before and during DAA therapy in order to identify potential biomarkers for the early diagnosis of liver tumor occurrence.

Despite the major limitation of our study—the small number of patients—we believe that our findings may contribute to the ongoing debate about DAAs and HCC occurrence and to provide new insight about circulating biomarkers in translational medicine. Long-term cohort studies are required to confirm our results on patients at risk of HCC development after DAA therapy.

## Figures and Tables

**Table 1:** Characteristics of cirrhotic patients enrolled in the study groups.

Characteristics	Study Group			*^§^ p*-Level
	Overall 40 Patients	Discovery 10 Patients	Validation 30 Patients	
Age, (years ± SD)	56.3 ± 10.3	59.4 ± 6.3	55.0 ± 11.1	0.07
Gender male/female, (% males)	26/14 (67.5)	6/4 (70)	20/10 (66.6)	0.84
BMI, (kg/m2 ± SD)	25.8 ± 3.1	27.3 ± 2.8	25.3 ± 3.1	0.08
Genotype HCV-1/non-1, (% HCV-1)	27/13 (67.5)	6/4 (60)	21/9 (70)	0.84
Child-Pugh score A5/A6-B7, (% A5)	30/10 (75)	8/2 (80)	22/8 (73.3)	1.0
Naive/Experienced, (% naive)	13/27 (32.5)	1/9 (10)	12/18 (40)	0.17
Sofosbuvir-based schedule, (%)	35/5 (87.5)	8/2 (80)	27/3 (90)	0.91
Ribavirin combination use, (%)	38/2 (95)	10/0 (100)	28/2 (93.3)	1.0
Treatment duration, (weeks ± SD)	18.3 ± 5.6	20.4 ± 5.1	18.6 ± 5.6	0.16
SVR/Relapse, (% SVR)	32/8 (80)	7/3 (70)	25/5 (83.3)	0.64
ALT (U/L ± SD)	114.9 ± 79.1	103.7 ± 87.6	110.2 ± 76.5	0.56
Albumin (mg/dL ± SD)	40.9 ± 3.4	41.1 ± 3.5	39.2 ±3.4	0.15
Bilirubin (μmol/L ± SD)	15.7 ± 7.1	14.5 ± 6.2	16.2 ± 8.3	0.36
PT (INR ± SD)	1.1 ± 0.08	1.0 ± 0.05	1.1 ± 0.09	0.76
PLTS (×10^9^/L ± SD)	164 ± 110	120 ± 70	179 ± 120	0.61
Alfa-fetoprotein (μg/L ± SD)	19 ± 32	17 ± 11	21 ± 36	0.14
HCC development, (%)	20 (50)	5 (50)	15 (50)	0.71
Time to HCC diagnosis from begin of DAAs (months ± SD)	8.9 ± 5.6	9.2 ± 5.1	8.8 ± 5.6	0.8

SVR: sustained virological response; HCC: hepatocellular carcinoma; DAAs: direct-acting antivirals. ^§^ T-test was applied for continuous variables and Chi-square Yates correct for categorical variables. Statistical comparison was performed between the discovery and validation cohorts.

**Table 2:** Expression levels of the miRNA candidates in the two groups determined by qRT-PCR.

miRNA	With HCC Mean Cq (95% CI)	With HCC Mean ΔΔCq (95% CI)	Without HCC Mean Cq (95% CI)	Without HCC Mean ΔΔCq (95% CI)
miR-483-5p	35.83 (35.19–36.47)	1.04 (0.62–1.47)	36.22 (35.60–36.83)	0.80 (0.51–1.10)
miR-1246	31.56 (31.06–32.06)	0.48 (0.34–0.63)	31.94 (31.50–32.38)	0.42 (0.24–0.61)
miR-1180-3p	33.41 (29.39–37.42)	0.37 (0.13–0.62)	32.77 (28.18–37.35)	0.18 (0.12–0.24)
miR-3197	36.00 (35.38–36.63)	0.33 (0.14–0.52)	34.46 (33.89–35.01)	0.80 (0.48–1.12)
miR-4443	27.75 (22.35–33.15)	0.14 (0.04–0.24)	26.71 (20.24–33.18)	0.10 (0.07–0.14)
miR-3178	33.70 (33.20–34.21)	2.61 (2.12–3.12	34.34 (33.82–34.85)	2.01 (1.48–2.53)
miR-1207-5p	31.07 (28.91–33.23)	0.62 (0.46–0.79)	32.02 (31.48–32.57)	0.81 (0.51–1.12)
miR-1228-3p	26.83 (26.59–27.07)	0.71 (0.42–0.99)	26.42 (26.14–26.71)	0.97 (0.71–1.2)
miR-4484	32.5 (31.58–33.43)	0.66 (0.43–0.88)	32.97 (32.56–33.38)	0.42 (0.33–0.51)

## Supplementary Materials

The following are available online at https://www.mdpi.com/2072-6694/11/11/1773/s1: Figure S1: Circulating miRNA expression in the 2nd and 3rd quantile, Figure S2: Circulating miRNA expression in the 4th and 5th quantile, Figure S3: Heatmap with the pseudocolor scale underneath of the differentially expressed miRNAs between T0 (red) and T1 (blue) in all samples. Figure S4: Gene enrichment analysis, Figure S5: Genes enriched in the “pathways in Cancer” term from KEGG database, Figure S6: Mir-3197 expression in the discovery cohort at T0 and T1, Table S1: Circulating miRNA differential expression between T0 and T1 in all samples, Table S2: Circulating miRNA differential expression between T0 and T1 in samples with HCC, Table S3: Circulating miRNA differential expression between T0 and T1 in samples without HCC, Table S4: Experimentally validated miRNA targets with “strong evidence”, Supplementary Materials and methods and Results, Supplementary file 1: MiRNA targets, Supplementary file 2: Enriched pathways, Supplementary file 3: Enriched pathways in miRNA targets with strong evidences
